# The green rice leafhopper, *Nephotettix cincticeps* (Hemiptera: Cicadellidae), salivary protein NcSP75 is a key effector for successful phloem ingestion

**DOI:** 10.1371/journal.pone.0202492

**Published:** 2018-09-05

**Authors:** Yukiko Matsumoto, Makoto Hattori

**Affiliations:** Institute of Agrobiological Sciences, National Agriculture and Food Research Organization, Owashi, Tsukuba, Ibaraki, Japan; Public Library of Science, UNITED KINGDOM

## Abstract

*Nephotettix cincticeps*, a prevalent rice pest, injects gelling and watery saliva into plant tissues during the sucking process. Certain components within the saliva are believed to interact with plant cellular constituents and play important roles in overcoming host plant defense responses. Based on our previous analysis of the salivary gland transcriptome and secreted saliva proteome of *N*. *cincticeps*, in this study, we analyzed the biological functions of salivary protein, NcSP75 (*N*. *cincticeps* salivary protein 75 kD). *NcSP75*, a salivary glands-specific gene, showed low similarities to any previously reported sequences. Knockdown of *NcSP75* by RNA interference (RNAi) reduced the longevity of treated nymphs to approximately half of the longevity of controls and caused severe developmental retardation. Furthermore, the knockdown of *NcSP75* decreased the survival rate of adults, and reduced the number of deposited eggs and hatched nymphs. Thus, the adverse effects caused by the knockdown of *NcSP75* were observed throughout the lifetime of *N*. *cincticeps*, when feeding on rice plants. In contrast, no reduction was observed in the survival rate of the knockdown of *NcSP75* adults fed on an artificial diet. Electrical penetration graph measurements taken from adult females feeding on rice plants showed a significantly shorter duration of phloem ingestion associated with the knockdown of *NcSP75* than the knockdown of the enhanced green fluorescent protein (EGFP). Furthermore, the total sugar content of the honeydew was lower when *NcSP75* was knocked down. These results suggest that the NcSP75 protein contribute to successful and sustainable ingestion from the sieve elements of rice plants. The NcSP75 protein of *N*. *cincticeps* can, accordingly, be considered as a key effector for establishing compatible interaction with rice plants and could be a potential target for controlling this species.

## Introduction

Many piercing-sucking insect species damage host plants by ingesting sap from vascular tissues; thus, causing substantial losses of crop yields worldwide [1–3]. Vascular feeders inject two types of saliva into plant tissues before and/or while sucking phloem sap: gelling saliva, whose components mainly form the salivary sheath surrounding the stylets inside the host plants [4, 5]; and watery saliva, whose role is considered to consist in suppressing and/or overcoming the defense responses of plants. Thus, various salivary components may play important roles in feeding on host plants [5]. To date, extensive data have accumulated on the salivary gland transcriptome and proteome of vascular tissue-feeding hemipteran species, including the transcriptomes of *Empoasca fabae* Harris [6] and *Nilaparvata lugens* (Stål) [7] and the proteome of *Acyrthosiphon pisum* (Harris) [8, 9]. Although numerous salivary genes and proteins have been identified, functionally, only a few of them have been characterized [10].

To elucidate the function of hemipteran salivary proteins, the RNA interference (RNAi) method has often been used. RNAi knockdown of certain salivary gland-expressed genes, such as *c002* and other functional genes in *A*. *pisum* and *N*. *lugens* [11–14] causes lethality and/or a decrease in the phloem sap feeding time, which suggests that these genes are candidate effectors. Furthermore, knockdown has led to the identification of genes involved in the formation of the salivary sheath, for example, the structural sheath protein (*shp*) in *A*. *pisum*, and the salivary sheath protein (*NlShp*) in *N*. *lugens* [15, 16].

The green rice leafhopper *Nephotettix cincticeps* (Uhler) (Hemiptera: Cicadellidae) is widely distributed in temperate regions of Asia. This insect damages rice plants by sucking sap and vectoring virus- and phytoplasma-induced diseases [17–19]. The female lays 100–200 eggs inside the leaf sheaths of the plant over a period of about 3 weeks [20, 21]. Nymphs hatch within 10 days to develop through five ecdyses into adults in about three weeks at ca 25°C [22]. Like other vascular feeders, this species uses specialized needle-like mouthparts called stylets to penetrate plant tissues and suck the sap. Electrical penetration graphs (EPGs) of this insect on rice revealed that it mainly ingested from phloem or xylem of the plant [23]. While probing and sucking, it discharges gelling and watery saliva into the plant tissues [4, 24, 25]. The saliva components may play crucial roles in countering the plant defense system for successful ingestion from the phloem.

In previous studies, we analyzed the sialotranscriptome and the proteome of secreted saliva in *N*. *cincticeps*, in which more than 50,000 contigs and 71 secreted proteins were identified [26, 27]. Approximately 70% of the contigs and 40% of the secreted proteins were found to be dissimilar to known proteins [26, 27]. To date, a few proteins, such as laccase (NcLac1S), beta-glucosidase, and calcium-binding protein (NcSP84), have been characterized in *N*. *cincticeps* [26, 28–31]. NcLac1S belongs to the multicopper oxidase family and the secreted protein is encoded by the salivary gland-specific gene *NcLac1S* [26, 29]. This protein may be involved in the solidification of gelling saliva [32] and/or polymerization of monolignols in rice plants [29]. As beta-glucosidase activity has been detected in the salivary glands and in the salivary sheaths, it might be involved in the hydrolysis of a salivary phenol glucoside, which is a step in the solidification of salivary components to form salivary sheaths [31, 33]. NcSP84, which contains an EF-hand motif, is the most abundant salivary protein in *N*. *cincticeps*, and is injected into the sieve element tubes of rice plants during feeding. It has been suggested that NcSP84 binds to calcium ions in penetrated sieve tubes, thereby suppressing occlusion responses at the ruptured sites [26, 30].

To understand the molecular functions of the unknown salivary protein, we preliminarily examined the silencing effect of several dozens of saliva-specific genes on offspring nymphs using the parental RNAi method. We found that the number of hatched nymphs from the females injected with double-strand (ds)*NcSP75* gene drastically decreased. *NcSP75* is a salivary gland-specific gene in *N*. *cincticeps*, which encodes a secreted protein in saliva containing 717 amino acid residues (GenBank accession number BAQ94503; [26]). Sialotranscriptome analyses have shown that *NcSP75* is a highly expressed contig (TsukubaH.comp13028_c0_seq1) [27]. However, the predicted amino acid sequence of NcSP75 showed low sequence similarities to known proteins and its function has yet to be clarified.

In the present study, we demonstrated that knockdown of *NcSP75* causes a decrease in the longevity, and severe developmental retardation, of *N*. *cincticeps* nymphs, as well as a decrease in the survival rate of adults, ultimately leading to a decrease in reproduction. Furthermore, NcSP75 was shown to be indispensable for normal ingestion from the phloem of rice plants.

## Materials and methods

### Insects

*Nephotettix cincticeps* was collected in Tsukuba, Ibaraki Prefecture, Japan, in 1993. A colony has since been maintained on rice seedlings in plastic cages (34 × 26 × 34 cm) in our laboratory, at 25°C and under a light: dark photoperiod regime of 16:8 h [28].

### Double-stranded (ds) RNA construction and injection

A 469-bp fragment of the *NcSP75* gene (GenBank accession number BAQ94503 [26]) was amplified by polymerase chain reaction (PCR) using the primers NcSP75_f1 and NcSP75_r1 (Table 1). Similarly, a 548-bp fragment of the enhanced green fluorescent protein (EGFP) gene derived from the plasmid pEGFP vector (Clontech Laboratories, Inc., Mountain View, CA, USA), was amplified using primers EGFP_f and EGFP_r (Table 1). The PCR products were ligated into a pGEM-T vector (Promega Corporation, Madison, WI, USA). To obtain single-stranded RNA (ssRNA) templates, the PCR products were further amplified to incorporate the T7 RNA polymerase promoter sequence from the plasmids using the following primers: T7 promoter_SP6 and NcSP75_f1, and T7 promoter_T7 and NcSP75_r1 for pGEM-*NcSP75*, and T7 promoter_SP6 and EGFP_r, and T7 promoter_T7 and EGFP_f for pGEM-*EGFP* (Table 1). The dsRNAs were prepared as described previously [34].

**Table 1:** Primers used in the study.

Primer	5′→ 3′		
**DsRNA**			
**NcSP75_f1**	**TCGGACTTTATTCAGCATTTGATA**		
**NcSP75_r1**	**GTTTTGCGAGGTCAGTTTTCTTAT**		
**EGFP_f** ^a^	**TGACCCTGAAGTTCATCTGCACC**		
**EGFP_r** ^a^	**CACGAACTCCAGCAGGACCAT**		
**T7 promoter_SP6** ^a^	**GGATCCTAATACGACTCACTATAGGGATTTAGGTGACACTATAGAATACT**		
**T7 promoter_T7** ^a^	**GGATCCTAATACGACTCACTATAGGGTAATACGACTCACTATAGGGCGA**		
**qPCR**			
		**Tm (degrees)**	**Amplicon length (bp)**
**NcSP75_f2**	**GTATCGACAGCCTCAACGAAG**	**58.6**	
**NcSP75_r2**	**ACTGAACGCCCTCTCTTGTCT**	**62.5**	***NcSP75*, 117**
**EF1_f** ^b^	**CAGTGAGAGCCGTTTTGAG**	**57.3**	
**EF1_r** ^b^	**AGGGCATCTTGTCAGAGGGC**	**63.5**	***EF1*, 143**
**Whole-mount *in situ* hybridization**			
**T7_NcSP75_f3**	**CTTAATACGACTCACTATAGGGCCAGCGTATCTATGATCTCAAC**		
**T7_NcSP75_r3**	**CTTAATACGACTCACTATAGGCGCTAGAGTTTCGCATTCAACTT**		
**NcSP75_f3**	**GCCAGCGTATCTATGATCTCAAC**		
**NcSP75_r3**	**CGCTAGAGTTTCGCATTCAACTT**		

^a^ Reference [35].

^b^ Reference [34].

The T7 RNA polymerase recognition site sequence is underlined.

The dsRNAs were injected using the system described by Tomizawa and Noda [35]. Insects were anesthetized on ice before injection. The glass capillary used for injection was inserted into the intersegmental region between the thorax and the abdomen on the ventral side of the insects. For both, *NcSP75* and *EGFP* knockdown, approximately 30 nL of dsRNAs (300 ng/μL) were injected into the insects.

### RNA interference (RNAi)

#### Nymphal RNAi

Third-instar nymphs were injected with *NcSP75* or *EGFP* dsRNA within 24 h after molting. Injected nymphs were maintained individually in glass tubes containing rice seedlings and monitored to determine survival rate and developmental stage. An untreated group (not injected control) was maintained under similar conditions. Nymphs were transferred to glass tubes containing new seedlings every 3–4 days. Survival and molting rates were recorded every day (*NcSP75*, n = 31; *EGFP*, n = 29; and untreated control, n = 30).

#### Adult RNAi

Adult females and males were injected with *NcSP75* or *EGFP* dsRNA within 24 h after eclosion. An untreated group was maintained under similar conditions. To estimate the effect of RNAi on female survival and reproduction, each treated female was paired with an untreated male for mating in a new glass tube containing rice plant seedlings after being maintained individually for a premating period of 4 days [36]. Each pair was transferred to a glass tube containing new rice seedlings every 2 days for 10 days or until death of the female. A new male was added when a male died. Females were allowed to lay eggs for 4–14 days after dsRNA injections. The number of eggs laid per female were counted 5–8 days after the beginning of oviposition by dissecting host plants under a stereomicroscope (*NcSP75*, n = 71; *EGFP*, n = 58; and untreated control, n = 59). The numbers are calculated from two independent experiments (S1 Fig). The numbers of hatched nymphs per female were also counted 8–14 days after the beginning of oviposition under a stereomicroscope (*NcSP75*, n = 18; *EGFP*, n = 18; and untreated control, n = 18).

To estimate the effect of RNAi on male survival on rice plants, males were maintained individually in glass tube containing rice plant seedlings and transferred to another glass tube containing rice seedlings every 3–4 days. The number of surviving individuals was recorded every day (*NcSP75*, n = 40; *EGFP*, n = 40; and untreated control, n = 37). The numbers are calculated from two independent experiments (S2 Fig).

### Quantitative real-time PCR (qPCR)

To confirm the effects of RNAi on nymphs and adult females and males, a time-course of expression analysis was performed by qPCR. Total RNA was purified from (i) whole bodies of nymphs at 2, 4, 6, and 8 days after being injected with dsRNA, and of untreated nymphs (nymphal RNAi; n = 6); and (ii) heads containing salivary glands of adult females and males at 2, 4, 8, and 14 days and 2, 4, and 6 days after being injected with dsRNAs, and of untreated adults (adult RNAi; n = 6), respectively, using RNeasy (Qiagen). Complementary DNA strands (cDNAs) were synthesized from 100 ng RNAs using random 6-mer primers and a PrimeScript RT reagent kit (Perfect Real Time) (Takara Bio Inc., Otsu, Japan) according to the methods described previously [34]. qPCR was performed using SYBR Green I Master Mix (Roche Diagnostics, Basel, Switzerland) in a Light Cycler 480 System (Roche Diagnostics) with the cycling parameters of 95°C for 5 min, followed by 50 cycles of 95°C for 10 s, 60°C for 20 s, and 72°C for 10 s. The data were analyzed by absolute quantification and normalized with the *N*. *cincticeps* elongation factor-1 gene (*EF-1*) gene (GenBank accession number AB836664; [35]). The pGEM-T vectors containing partial *EF-1* or *NcSP75* sequences were used as gene-specific standards. The primers used for qPCR were NcSP75_f2 and NcSP75_r2 for *NcSP75* and EF1_f and EF1_r for *EF-1* (Table 1).

### Survival rate on an artificial diet

Adult males were injected with *NcSP75* or *EGFP* dsRNAs within 24 h after eclosion. To examine the survival rate, 3–5 males were confined in a small plastic dish containing an artificial diet that was covered with two layers of stretched-parafilm M (Bemis, WI, USA). The artificial diet consisted of 5% sucrose and 0.005% riboflavin in distilled water, which was filtered through a 0.22-μm syringe filter (Millipore, MA, USA). A filter paper (Advantec, Tokyo, Japan) was placed on the bottom of the dish to absorb the honeydew droplets, thereby preventing insects from sticking to the dish. The artificial diet was changed every 2–3 days. The number of surviving individuals was recorded every day (*NcSP75*, n = 67 and *EGFP*, n = 66). The numbers are calculated from two independent experiments (S3 Fig).

### Electrical penetration graph (EPG)

To monitor the feeding behavior of adult females injected with *NcSP75* or *EGFP* dsRNA, a Giga-8 DC recording system was used (Wageningen Agricultural University, Wageningen, Netherlands; http://www.epgsystems.eu/). Four days after treatments, females were anesthetized with CO_2_. One end of a gold wire (18 μm in diameter, 3–5 cm length) was attached to the dorsal thorax of the insect using silver glue. The other end of the gold wire was attached to the EPG probe. After recovering from anesthesia, individual insects were allowed to settle on a rice plant (approximately 3 weeks old). A rooted rice plant was placed into a plastic pot containing tap water. The electrode (2 mm diameter, 10 cm length) was immersed in the tap water. The insects and the rice plants were subsequently placed in a Faraday cage and EPG recording was carried out for 18–24 h in females treated with *NcSP75* (n = 21) and *EGFP* (n = 20) dsRNAs. The average duration of the respective waveforms was calculated for non-penetration (Non), salivation (S), trial ingestion of phloem (T_IP), ingestion of phloem (IP), ingestion of xylem (IX), and ingestion of other tissues (IO) [23, 37, 38]. The data were processed using Stylet^+^ software (Wageningen Agricultural University).

### Honeydew analysis

To examine the effect of *NcSP75* RNAi on phloem feeding by *N*. *cincticeps*, the total sugar content of honeydew was determined using the anthrone method [39]. Adult females were injected with *NcSP75* and *EGFP* dsRNAs within 24 h of eclosion. An untreated group was maintained under similar conditions. After 4 days, three insects were confined in a parafilm M-sachet (approximately 10 cm × 5 cm) attached to the leaf sheath of an approximately 3-week-old rice plant. The honeydew produced for 24 h was collected and stored at −20°C until further analysis. Twenty replicates were performed for each treatment. The anthrone reagent 150 μL (100 mg anthrone per 130 mL of cold 75% sulfuric acid) was added to 15 μL of honeydew and boiled for 10 min. After cooling, the samples were transferred to a 96-well microplate and absorbance was measured at 620 nm using a SpectraMax 250 microplate reader (Molecular Devices, Tokyo, Japan). Total sugar content per insect was estimated against a glucose standard curve.

### Whole-mount *in situ* hybridization

To obtain templates of digoxigenin (DIG)-labeled RNA, the PCR products were further amplified to incorporate the T7 RNA polymerase promoter sequence from the plasmid pGEM-*NcSP75* using the following primers: T7 promoter_NcSP75_f3 and NcSP75_r3 (for the sense strand), and NcSP75_f3 and T7 promoter_NcSP75_r3 (for the antisense strand) (Table 1). PCR conditions were as follows: initial denaturation at 94°C for 2 min followed by 5 cycles at 94°C for 30 s, at 56°C for 30 s, and at 72°C for 1 min; 25 cycles at 94°C for 30 s, at 60°C for 30 s, and at 72°C for 1 min. Final extension was at 72°C for 5 min. The PCR products were purified using a Wizard SV Gel and PCR Clean-Up System (Promega). DIG-labeled RNAs were synthesized using a DIG-RNA labeling kit (Roche Diagnostics) following the standard procedure. DIG-labeled RNAs (401 bp) were stored at −20°C until use. Whole-mount *in situ* hybridization of salivary glands was carried out using the protocols described by Mitsumori and Noji [40]. Adult females were injected with *NcSP75* and *EGFP* dsRNA within 24 h of eclosion. Four days later, the females were dissected, and their heads were fixed with 4% paraformaldehyde in PBSTx (phosphate-buffered saline containing 0.1% Triton X-100). The salivary glands were eviscerated from the head 4 h later and treated with 20 μg/mL proteinase K for 15 min at 20°C. Hybridization was performed with 400 ng/mL of DIG-labeled RNA at 70°C overnight. Specific hybridization was detected with a 1000-fold dilution of anti-DIG-AP, Fab fragments (Roche). Coloring reactions were carried out with a 50-fold dilution of NBT/BCIP stock solution (Roche). Salivary glands were observed under a binocular microscope.

### Statistical analysis

Statistical analyses were performed using JMP, ver. 5.1 (SAS Institute Inc. JMP User’s Guide, Version 5.1, 2003). Survival rates were analyzed using the log-rank test with Bonferroni correction. The lifespan, duration of each instar, number of deposited eggs and hatched nymphs were analyzed using one-way analysis of variance (ANOVA) with Bonferroni correction. The transcript levels of *NcSP75* and *EF-1* were analyzed using the Tukey–Kramer (HSD) test. The percentage of the EPG duration time, honeydew content and total sugar content of honeydew were analyzed using the Mann–Whitney test with Bonferroni correction.

## Results

### Effects of RNAi on the survival and developmental rates of nymphs

**Fig 1:**
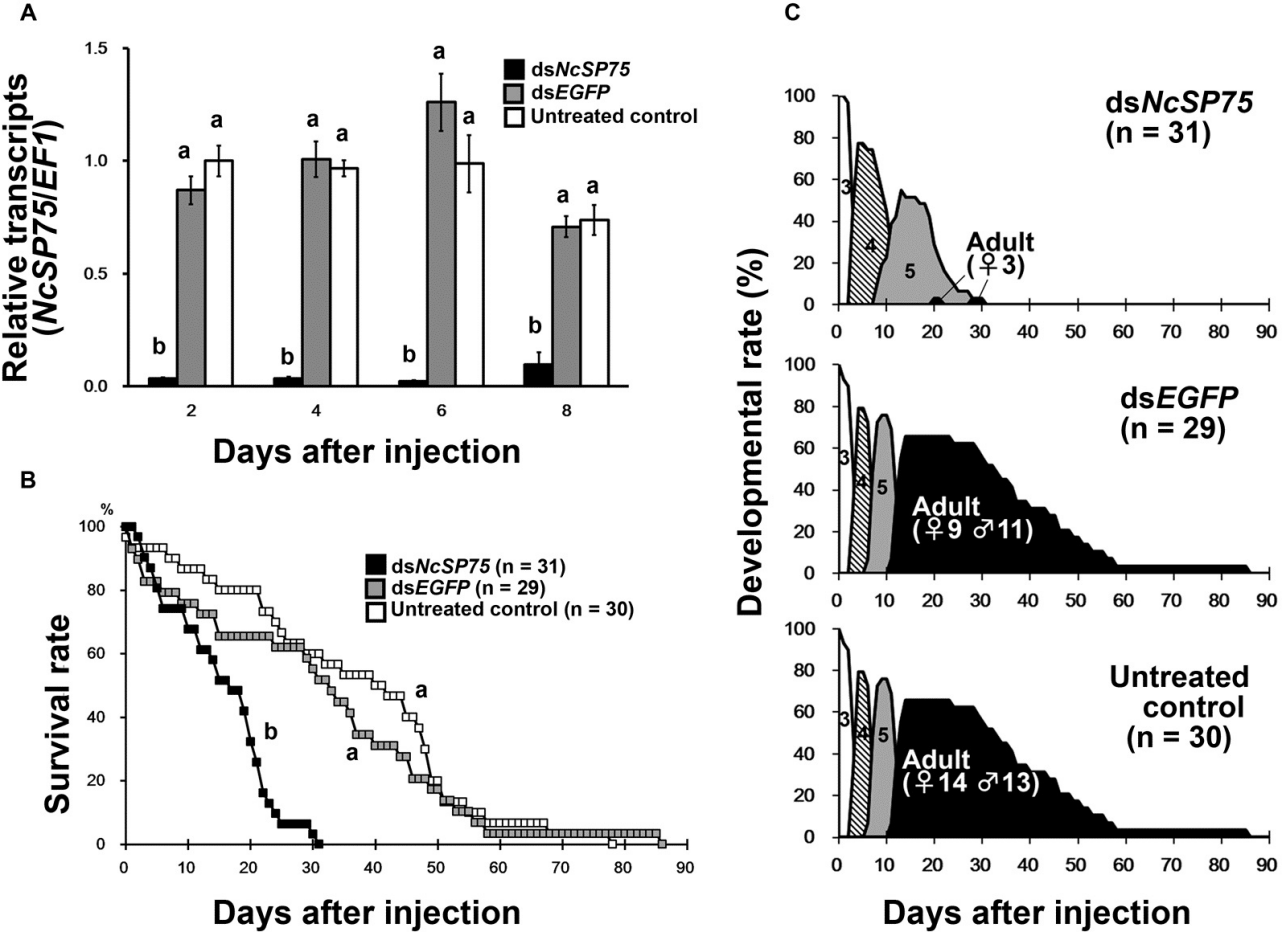
RNA interference of *NcSP75* in *Nephotettix cincticeps* nymphs. (A) Relative transcript levels of *NcSP75* in nymphs. Total RNA was extracted from the whole bodies of nymphs (n = 6) injected with *NcSP75* (black) and *EGFP* (gray) dsRNAs and untreated controls (white) at 2, 4, 6, and 8 days after injection. Elongation factor-1 (*EF-1*) was used as an internal control. The bars indicate standard errors (SE). The values are expressed as a ratio to the mean of the untreated controls after 2 days. Different letters indicate significant differences on the same day (p < 0.05). (B) Survival rate of 3rd-instar nymphs after injection of dsRNA. Survival rate and developmental stage of 3rd-instar nymphs after injection of dsRNA. *NcSP75* (black), *EGFP* (gray) dsRNAs and untreated control (white) were injected into 3rd-instar nymphs within 24 h of molting (day 0). The number of nymphs is shown in parentheses. Different letters indicate significant differences (p < 0.01). (C) Developmental stage and survival rate of nymphs in (B). The numbers 3 (white), 4 (hatched line), and 5 (gray) indicate nymph instar. Adults are indicated with black.

Third-instar nymphs were injected with dsRNA within 24 h of molting. The expression of *NcSP75* mRNA was significantly low in *NcSP75* dsRNA-injected (ds*NcSP75*-treatment) nymphs compared with ds*EGFP*-treated and untreated control nymphs at 2–8 days after dsRNA injection (Fig 1A, p < 0.05). The survival rate of ds*NcSP75*-treated nymphs was significantly decreased compared with that of the ds*EGFP*-treated and untreated nymphs (Fig 1B, p < 0.01). Approximately 50% of the individuals in the ds*NcSP75-* and ds*EGFP*-treated and untreated groups died on days 17, 33, and 45 after dsRNA injection, respectively. More than 90% of the individuals in the ds*NcSP75-* and ds*EGFP*-treated and untreated groups died on days 24, 56, and 61 after injections, respectively (Fig 1B). Ds*NcSP75*-treated nymphs showed apparent retardation of development (Fig 1C). From nymphs treated at the 3rd-instar, 4th-instar stage emerged at 3–14, 3–9, and 2–7 days after injection with ds*NcSP75* and ds*EGFP* or untreated controls, respectively. The 5th-instar nymphs appeared after 8–30, 6–14, and 5–14 days of injection with ds*NcSP75* and ds*EGFP* and untreated controls, respectively (Fig 1C), and adults emerged on day 20, 11, and 10 after injections with ds*NcSP75* and ds*EGFP*, and untreated controls, respectively. Most of the ds*NcSP75*-treated nymphs died during the 4th- and 5th-instar stages (Fig 1C). Only three females reached the adult stage (9.7%, 3/31), but lived for only a few days after eclosion. In contrast, for the ds*EGFP*-treated and untreated groups, 69.0% (20/29) and 90.0% (27/30) of insects, respectively, grew to the adult stage and lived for approximately 1 month (Fig 1C and S1 Table). The lifespan of ds*NcSP75*-treated nymphs was approximately half that of the ds*EGFP*-treated and untreated nymphs (S1 Table, p < 0.01). The duration of the 3rd-instar stage was significantly longer (0.79–0.90 days) for both ds*NcSP75* and ds*EGFP* treatment groups compared with the untreated group (S1 Table, p < 0.01). The duration of the 4th- and 5th-instars of ds*NcSP75*-treated nymphs was approximately twice as long as the corresponding durations in the ds*EGFP*-treated and untreated nymphs (S1 Table, p < 0.01).

### Effects of RNAi of NcSP75 on adult females

**Fig 2:**
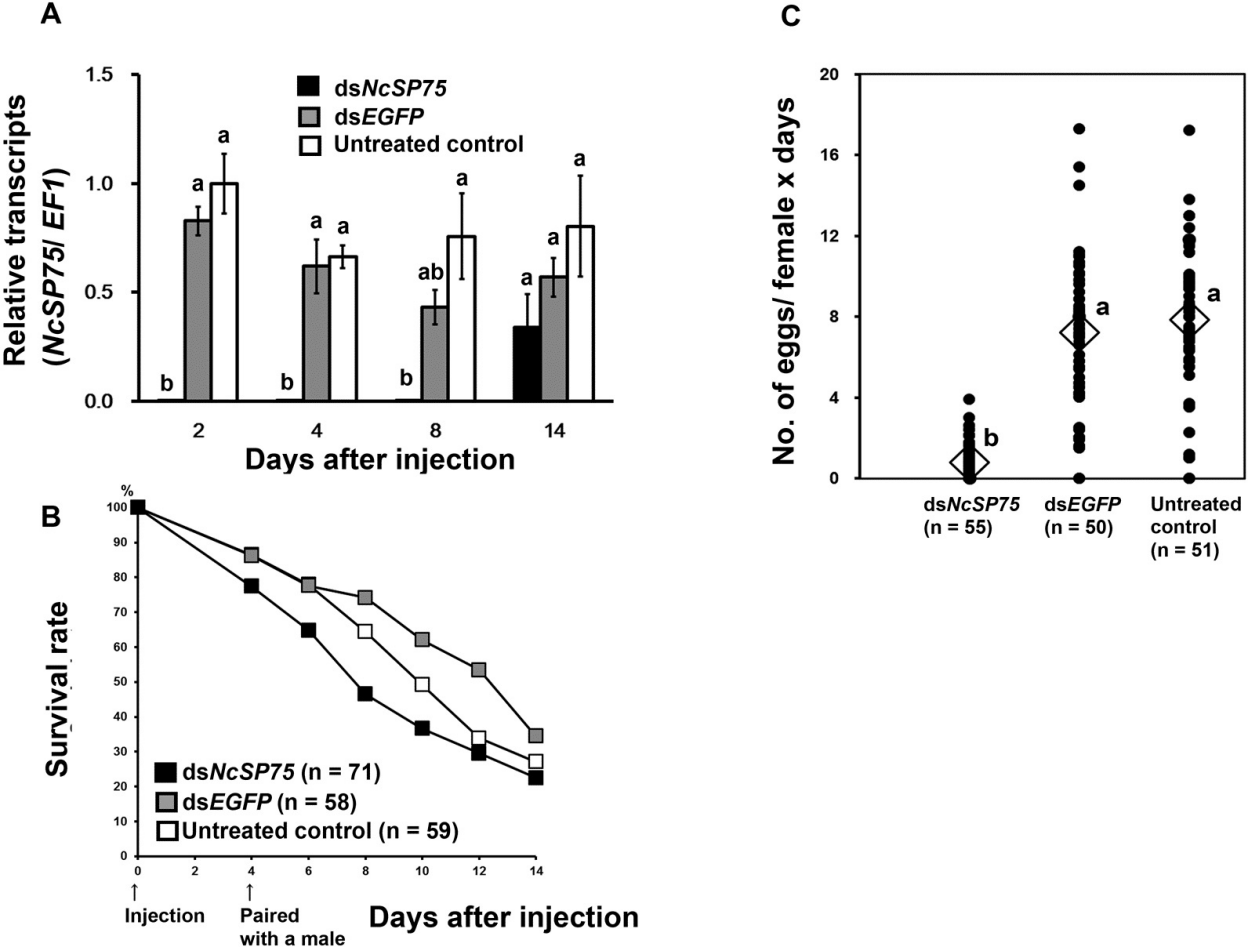
RNA interference of *NcSP75* in *Nephotettix cincticeps* adult females. (A) Relative transcript levels of *NcSP75* in adult females. Total RNAs were extracted from adult female heads (n = 6) containing salivary glands on days 2, 4, 8, and 14 after injection with *NcSP75* (black) and *EGFP* (gray) dsRNAs and untreated controls (white). Elongation factor-1 (*EF-1*) was used as an internal control. The values are expressed as a ratio to the mean of the untreated control after 2 days. Bars indicate standard errors (SE). Different letters indicate significant differences on the same day (p < 0.05). (B) Survival rates of adult females. *NcSP75* (black) and *EGFP* (gray) dsRNAs and untreated controls (white) were injected within 24 h of eclosion (day 0). The number of females is shown in parentheses. Each female was paired with a male for 4–14 days. No significant differences were found. (C) The number of eggs laid by surviving females was counted by dissecting the leaf sheath of the rice seedlings. Dot symbols indicate the mean number of eggs laid by individual females per day. Diamond symbols indicate the mean number of eggs laid. Different letters next to diamond symbols indicate significant differences (p < 0.01). The number of mated females is shown in parentheses. Twenty-three of 55 ds*NcSP75*-treated, two of 50 ds*EGFP*-treated, and two of 51 untreated females failed to lay any eggs.

To determine the effect of *NcSP75* RNAi on reproduction, adult females were injected with ds*NcSP75* within 24 h of eclosion. At 2, 4, and 8 days after dsRNA injection, the expression levels of *NcSP75* had decreased to 0.2–0.5% of those of the untreated group (Fig 2A, p < 0.05). At 14 days after dsRNA injection, no significant differences were observed in the *NcSP75* transcript levels among the experimental groups.

The survival rate of females injected with ds*NcSP75* tended to be lower than that of the ds*EGFP* and untreated groups; however, no significant differences on survival rate were detected among the treatments (Figs 2B and S4A). The number of eggs laid on the rice seedlings per day by the ds*NcSP75*-treated females was lower (mean ± SE, 0.79 ± 0.13) than that laid by ds*EGFP*-treated (7.22 ± 0.54) and untreated (7.86 ± 0.51) females (Fig 2C, p < 0.01). As many as 41.8% of the ds*NcSP75*-treated females laid no eggs (23/55) throughout the experiment, whereas less than 4.0% of ds*EGFP*-treated (2/50) and untreated (2/51) females failed to lay eggs. The number of nymphs hatched from eggs produced by the ds*NcSP75*-treated females (0.74 ± 1.01) was also lower than that from ds*EGFP*-treated (4.54 ± 2.89) and untreated (5.10 ± 2.30) females (S4B Fig, p < 0.01).

### Effects of RNAi on the survival rate of adult males

**Fig 3:**
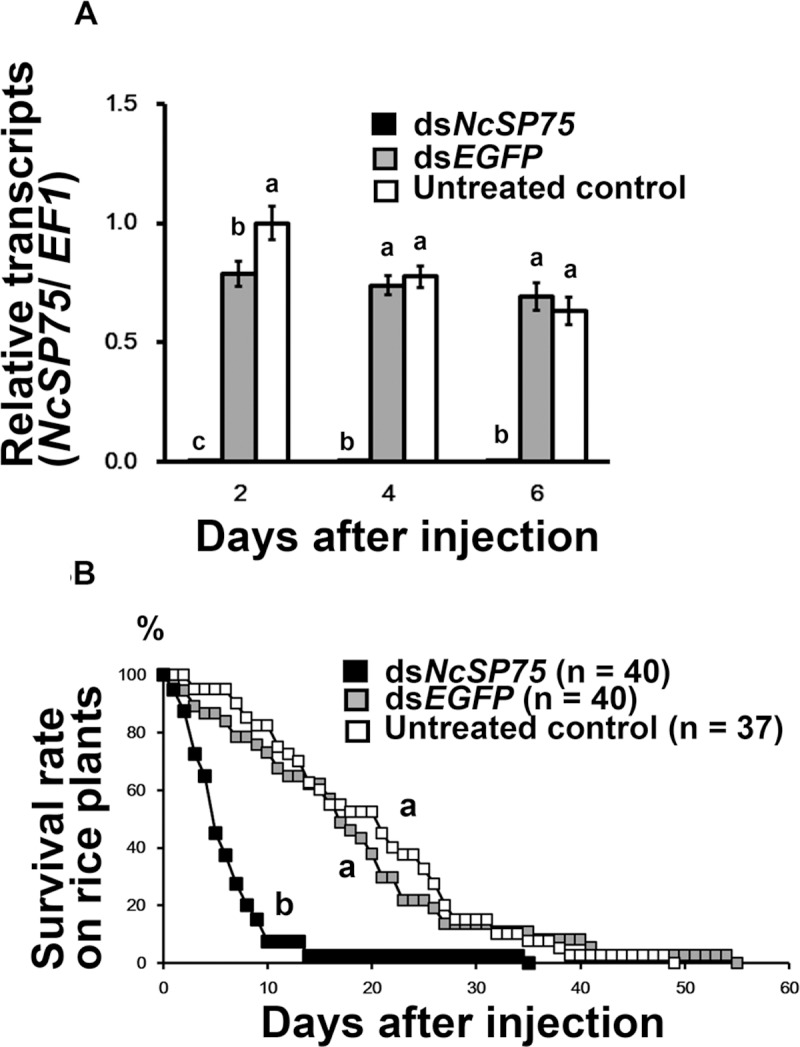
Effect of RNA interference on the survival rate of *Nephotettix cincticeps* adult males on rice seedlings. (A) Relative transcript levels of *NcSP75* in adult males. Total RNAs were extracted from adult male heads (n = 6) containing salivary glands on days 2, 4, and 6 after injection with *NcSP75* (black) and *EGFP* (gray) dsRNAs and untreated controls (white). Elongation factor-1 (*EF-1*) was used as an internal control. The values are expressed as a ratio to the mean of the untreated control after 2 days. Bars indicate standard errors (SE). Different letters indicate significant differences on the same day (p < 0.05). (B) Survival rate of males. Ds*NcSP75* (black), ds*EGFP* (gray), and untreated controls (white). DsRNAs were injected into adult males within 24 h of eclosion (on day 0). Different letters indicate significant differences (p < 0.01). The number of males is shown in parentheses.

**Fig 4:**
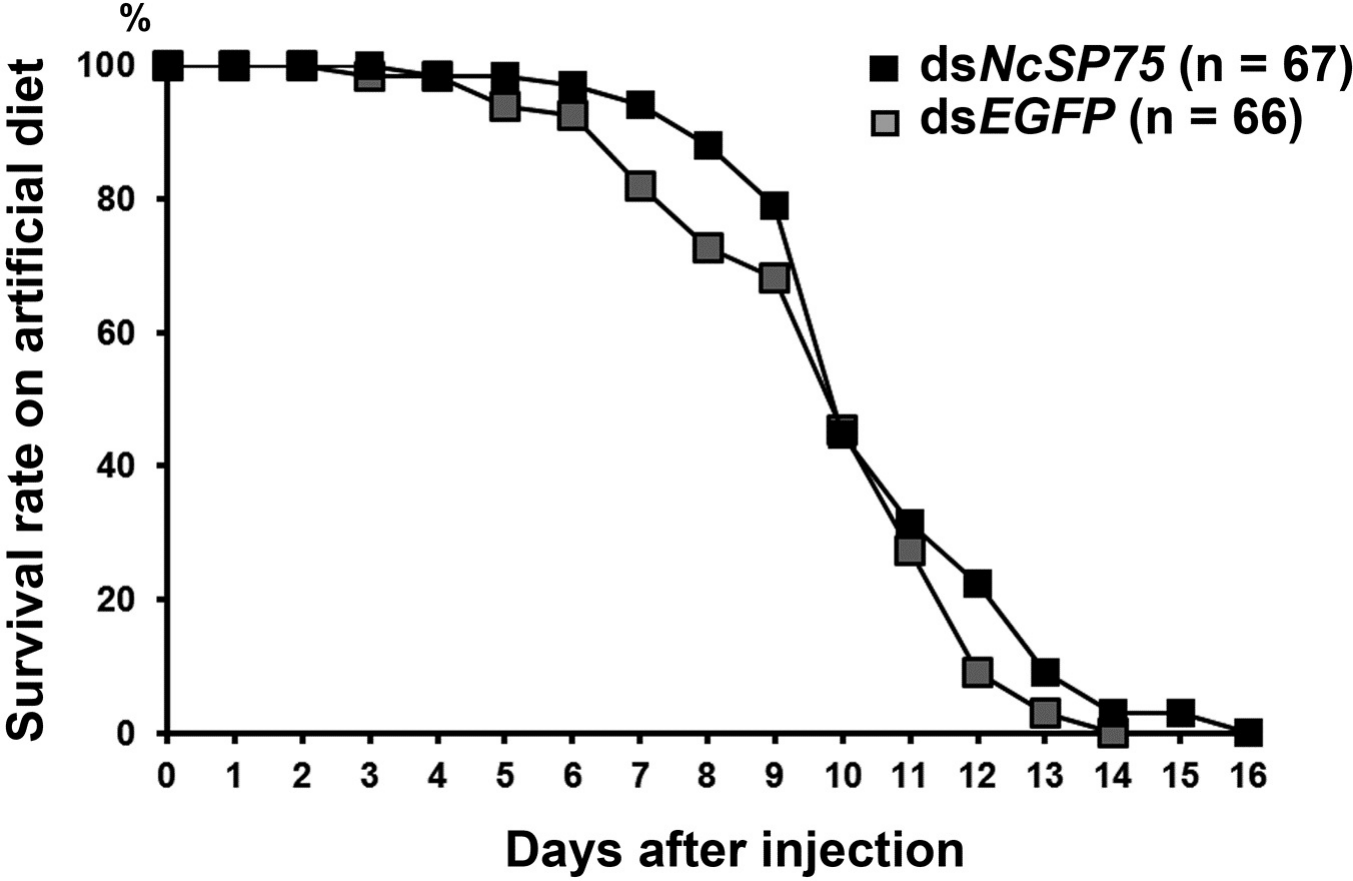
Effect of RNA interference on the survival rate of *Nephotettix cincticeps* adult males fed on an artificial diet. *NcSP75* (black) or *EGFP* (gray) dsRNA was injected into the adult males within 24 h of eclosion (day 0). Insects were provided an artificial diet (5% sucrose and 0.005% riboflavin in distilled water). No significant difference was found between the ds*NcSP75* and ds*EGFP* treatments.

The expression of *NcSP75* in ds*NcSP75*-treated adult males decreased to only 0.3–0.5% of the corresponding value in ds*EGFP*-treated and untreated males at 2–6 days after injection (Fig 3A, p < 0.05). The survival rate of ds*NcSP75*-treated adult males on rice seedlings sharply decreased in comparison to ds*EGFP*-treated and untreated males (Fig 3B, p < 0.01). Approximately 50% of the ds*NcSP75*-treated males had died after 5 days, and 92.5% of males had died after 10 days (Fig 3B). In the case of the ds*EGFP*-treated and untreated groups, half of the males had died after 21 days and 17 days, respectively, and more than 90% had died at 35 and 36 days after injection, respectively. The mean lifespan of the ds*NcSP75*-treated adult males was approximately one-third that of the ds*EGFP*-treated and untreated adult males (S2 Table, p < 0.01). In contrast, when fed on an artificial diet, there was no significant difference in the survival rates between ds*NcSP75* and ds*EGFP* treatments (Fig 4).

### Effects of RNAi on feeding behavior

**Fig 5:**
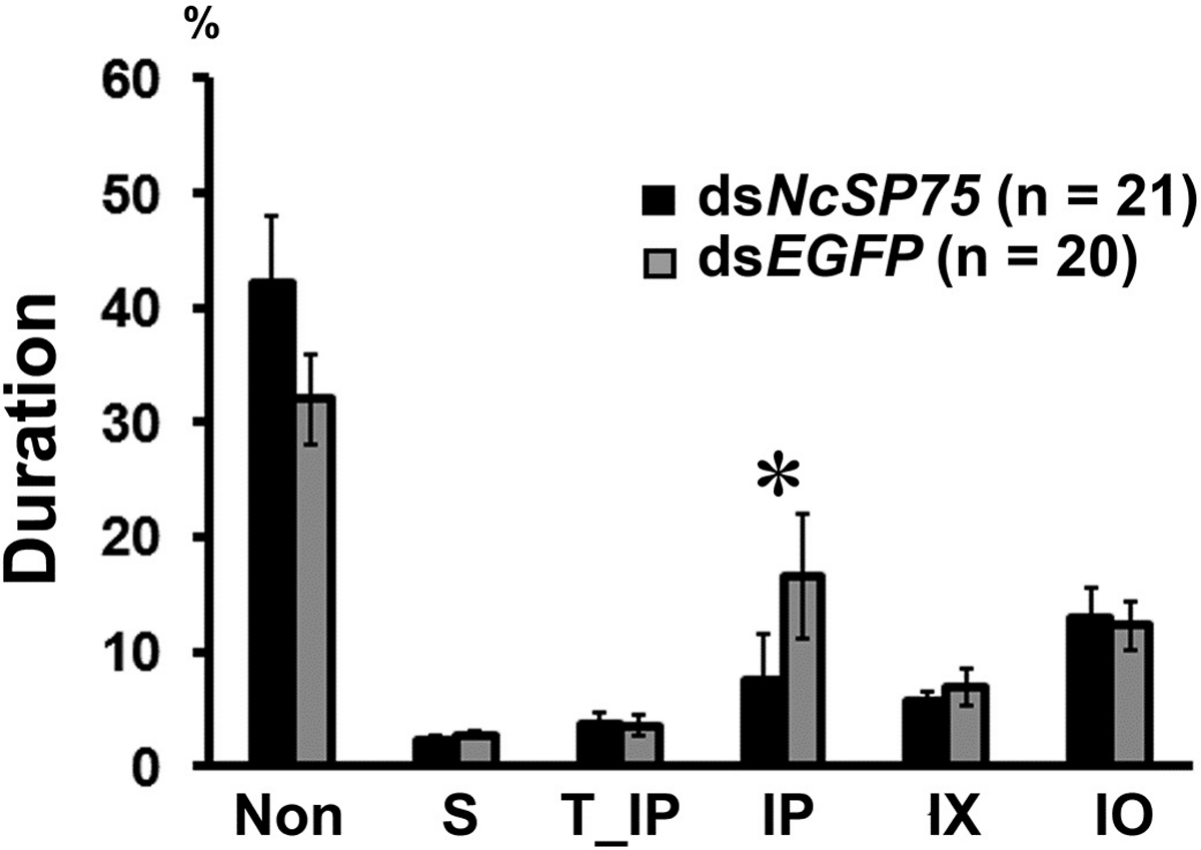
Electrical penetration graph (EPG) results for the *Nephotettix cincticeps* adult females injected with *NcSP75* and *EGFP* dsRNAs. The waveforms were classified as non-penetration (Non), salivation (S), trial ingestion of phloem (T_IP), ingestion of phloem (IP), ingestion of xylem (IX), and ingestion of other tissues (IO). EPG recording was carried out for 18–24 h in adult females at 4 days after injection with dsRNAs. Bars indicate standard errors (SE). An asterisk indicates a significant difference (*p < 0.05) between ds*NcSP75* (black, n = 21) and ds*EGFP* (gray, n = 20) treatments.

EPG recording was performed on adult females 4 days after injection with ds*NcSP75* or ds*EGFP* dsRNA. The waveform patterns were classified into six behavioral events based on Kawabe [23]: non-penetration (Non), salivation (S), trial ingestion of phloem (T_IP), ingestion of phloem (IP), ingestion of xylem (IX), and ingestion of other tissues (IO). Ds*NcSP75* treatment reduced the average duration of IP to 45.7% relative to the ds*EGFP* treatment (Fig 5, p < 0.05). No significant differences were observed for the duration of any other behavioral event, including xylem ingestion.

### Effect of RNAi on honeydew

**Fig 6:**
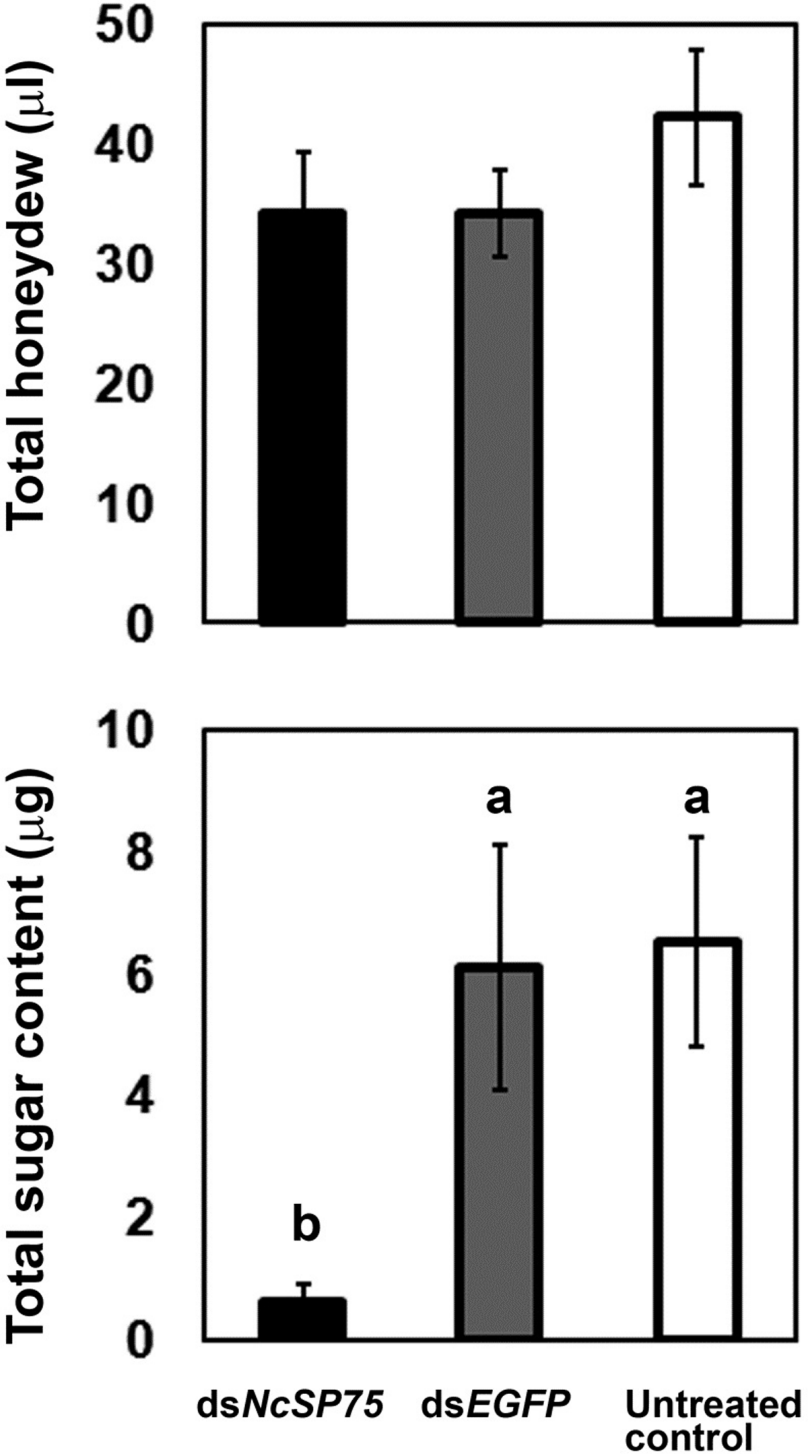
Effect of RNA interference on the honeydew of adult females of *Nephotettix cincticeps*. Twenty samples (three adult females per sample) were examined for each treatment for 24 h. Bars indicate standard errors (SE). No significant differences were found in total honeydew amount (upper panel). Different letters indicate significant differences in total sugar content in honeydew (p < 0.01) (lower panel). Total sugar content was estimated against a glucose standard curve.

Honeydew was collected using the sachet method from ds*NcSP75*-treated, ds*EGFP*-treated, and untreated adult females at 4 days after dsRNA injection. No significant differences were found in the total honeydew amount among the three treatments (Fig 6, upper panel). However, total sugar content in the honeydew was considerably lower (9.2–9.8%) in the ds*NcSP75* treatment group than in the ds*EGFP*-treated and untreated groups (Fig 6, lower panel, p < 0.01).

### Localization of *NcSP75* mRNA in the salivary glands

**Fig 7:**
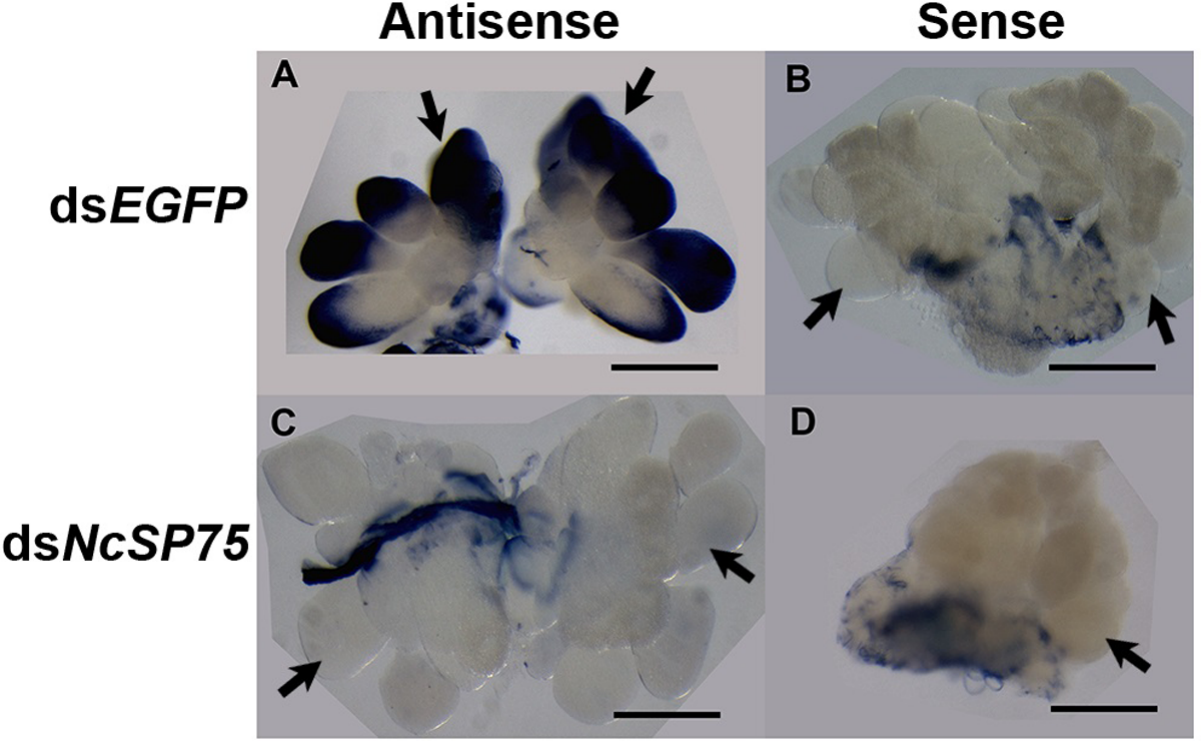
Expression of *NcSP75* mRNA in the salivary glands of *Nephotettix cincticeps*. The salivary glands were dissected from adult females injected with either ds*EGFP* (A and B) or ds*NcSP75* (C and D). *In situ* hybridization was performed with *NcSP75* antisense (A and C), or sense (B and D) probes. Arrows indicate some of the type III cells. The bar represents 0.2 mm. The cuticle of the trachea shows nonspecific staining.

The *NcSP75* gene is specifically expressed in salivary glands [26]. The expression site of *NcSP75* mRNA in the salivary glands was examined using adult females. In *N*. *cincticeps*, the principal salivary glands are composed of six cell types (I–VI) [41]. Whole-mount *in situ* hybridization using an *NcSP75*-antisense probe positively stained type III cells in the salivary glands of *EGFP*-treated females (Fig 7A), which were arranged radially like six petals [41], although nonspecific staining was observed in the cuticle of the trachea. In contrast, in the salivary glands of ds*NcSP75*-treated females, the signal was extremely weak (Fig 7C). No signal was observed with a sense probe for either ds*EGFP*- or ds*NcSP75*-treated females (Fig 7B and 7D).

## Discussion

*N*. *cincticeps* penetrates mesophyll or parenchyma cells with its stylet to reach the vascular bundles, whereas *A*. *pisum* and the whitefly *Bemisia tabaci* (Gennadius) penetrate the epidermis with their stylets and intercellularly probe parenchyma cells before reaching the phloem [42–46]. Thus, *N*. *cincticeps* likely encounters more defensive chemicals, which are stored in vacuoles and in the intercellular apoplast, than the other species. Furthermore, *N*. *cincticeps* is an oligophagous hemipteran species that sucks sap from rice and some other poaceous plants. Therefore, unique salivary components in *N*. *cincticeps* might have evolved to counteract the constitutive and inducible defenses of various host plants during the feeding process. Many of the salivary protein genes, including *NcSP75*, are species-specific and specifically expressed in salivary glands [26, 27].

Gene knockdown of the *NcSP75* salivary gene of *N*. *cincticeps* by RNAi, markedly reduced survival rate and caused severe developmental retardation in nymphs on rice plants (Fig 1B and 1C). In nymphal RNAi of *NcSP75*, most of the injected 3rd-instar nymphs reached the 4th-instar stage. However, in both, ds*NcSP75* and ds*EGFP* treatments, 3rd-instar nymphs required in average 0.79–0.90 more days to develop into the 4th-instar, compared with the untreated group (S1 Table). This delay in the molt to the nymph 4th-instar was probably due to piercing by a glass capillary used for dsRNA injection [35]. The durations of the 4th- and 5th-instar stages were considerably longer in the ds*NcSP75-*treated nymphs than in the ds*EGFP*-treated or the untreated nymphs. Thus, the RNA knockdown effect was maintained across nymphal stages. Eventually, most of the ds*NcSP75*-treated nymphs died before reaching the adult stage (Fig 1C and S1 Table). The developmental retardation and survival rate reduction were observed throughout the entire experimental period. Presumably, some harmful effect caused during NcSP75 knockdown in the early- to mid-stages, may gradually accumulate and cannot be overcome, even if the gene silencing effect becomes weak. The injection of ds*NcSP75* in adults considerably reduced their survival rate; whereas surviving females laid fewer eggs (Figs 2C and 3B), resulting in a decrease in the number of hatched nymphs (S4B Fig).

It is a well-known fact that *N*. *cincticeps* nymphs and adults exhibit developmental retardation and a decrease in survival rate or reproduction when reared on resistant varieties of rice, because ingestion from phloem sap is inhibited [23, 47]. When reared on resistant varieties of rice plants, the longevity of adult males was much shorter (5.3–19.0 days, i.e., 49.4–74.6%) than longevity of adult females (7.1–27.8 days) [47]. The number of eggs laid on plants of resistant rice varieties decreased to 0–20.3%, compared to controls [47]. These data suggest that the inhibition of phloem sap ingestion affected the survival of males more than the survival of females; furthermore, it affected the reproductive ability of surviving females. Adult females seem to store much more nutrition than males at times of emergence, as females need to lay eggs. Accordingly, in the case of food shortage, in females, the nutrients for development of ovaries are probably allocated to survival. Ds*NcSP75*-treated insects showed a phenotype similar to that of insects reared on resistant rice plants (Figs 1B, 1C, 2C and 3B). Thus, a similar phenomenon may apply to ds*NcSP75*-injected females and males.

Unlike the case of the rice plants, no knockdown effects on the survival rates of adult males fed on an artificial diet were detected in the ds*NcSP75* treatment group (Fig 4). Thus, it is suggested that NcSP75 should be indispensable for feeding from phloem of rice plants. NcSP75, a secreted saliva protein, is possibly injected into plant tissue [26], thereby, overcoming certain types of defense response in rice plants. These results suggest that the NcSP75 protein functions as an effector.

EPG analysis of feeding behavior also showed that ds*NcSP75*-treated insects ingested from the phloem for a significantly shorter period but fed normally from xylem and other tissues (Fig 5). A vascular feeder, *N*. *cincticeps* excretes two types of honeydew: One contains rich sugar and amino acids, and the other has no sugar and only a small amount of amino acid [48]. Each honeydew is considered to be derived from the phloem and the xylem, respectively, because only phloem sap contains sucrose, which was present at 17–25% (w/v) [49]. Although the amount of honeydew was not significantly different among the treatments, the total sugar content was significantly reduced in the ds*NcSP75*-treatment group (Fig 6). These results also indicate inhibition of phloem sap feeding in the ds*NcSP75*-treatment group. Thus, these results suggest that the NcSP75 protein contributes to successful and sustainable ingestion from the phloem of rice plants.

EPG data suggested that ds*NcSP75*-treated insects ingested sap from the phloem to some extent (Fig 5) but excreted little sugar in honeydew (Fig 6). A small amount of nutrient including sugar ingested by the ds*NcSP75*-treated insects may be efficiently utilized or consumed in their bodies, resulting in little amount of sugar in honeydew.

In hemipteran species, the RNAi method has been used to demonstrate that certain salivary proteins are involved in their survival on host plants. In *A*. *pisum*, the knockdown of *C002* or *Armet* genes significantly reduced survival rate and sieve-element feeding time on plants [11, 12, 14]. Simultaneous knockdown of the *ACE1* and *ACE2* genes, which belong to the M2 metalloprotease family, also caused reduced survival of *A*. *pisum* on plants [13], but increased their sieve-element feeding time, in contrast to the knockdown of *C002* and *Armet* genes. ACEs are inferred to digest plant peptide hormones, such as systemin, or other signaling peptides that induce plant immune reactions [13]. In *N*. *lugens*, knockdown of the salivary gland-specific genes *salivap-3*, *NlShp*, *ANX-like5*, *NlSEF1*, and *NlMul* has been shown to decrease survival rate when fed on rice plants and to cause a reduction in ingestion time from phloem sap [15, 50–52]. It has been observed that *salivap-3*, *NlShp* and *NlMul* are involved in the formation of normal salivary sheaths. NlSEF1, a calcium binding protein with an EF-hand motif, is suggested to prevent plugging of sieve elements [51]. *NcSP75* knockdown in *N*. *cincticeps* resulted in a phenotype similar to that of insects with knockdown of some of the aforementioned salivary genes. However, NcSP75 does not contain specific motifs related to calcium-binding activity, and *NcSP75* knockdown insects produced normal salivary sheaths (data not shown). Although the precise function of NcSP75 remains unclear, this salivary protein might suppress a certain signal pathway leading to phloem occlusion, thereby enabling continued successful feeding from phloem. In rice plants infested by *N*. *lugens*, callose deposition in the sieve tubes is induced by insect attack; thereby, causing sieve tube occlusion [53]. In resistant varieties of rice, callose deposition was more abundant and longer-lasting than in susceptible varieties, which prevented *N*. *lugens* from feeding from phloem sap [53, 54]. Therefore, NcSP75 might be involved in suppressing callose deposition in sieve tube.

*NcSP75* mRNA was detected in the type III cells of the principal salivary glands (Fig 7A). In these cells with many canaliculi and typical alveolate features, *NcSP84*, alpha-glucosidase, and beta-glucosidase are also localized [30, 33, 41]. As no transmembrane domain was detected in NcSP75, except in the predicted signal peptide, this protein may be secreted from and stored in type III cells along with other salivary proteins.

## Conclusions

In this study, we demonstrated that NcSP75 is a salivary effector protein in *N*. *cincticeps* that is indispensable for establishing compatible interaction with rice plants for feeding from phloem vessels. This molecule could be used as a promising target to develop control measures for *N*. *cincticeps*. Further studies are needed to elucidate the details of its biochemical function.

## Supporting information

S1 Fig Effect of RNA interference on the survival rate of *Nephotettix cincticeps* adult females (see Fig 2B). Survival rates of adult females. Two independent experiments were performed. Fig 2B is the sum of these data. Ds*NcSP75* (black), ds*EGFP* (gray), and untreated controls (white). In the 1st experiment (solid line), Ds*NcSP75* (n = 41), ds*EGFP* (n = 29), and untreated controls (n = 29). In the 2nd experiment (dashed line), Ds*NcSP75* (n = 30), ds*EGFP* (n = 29), and untreated controls (n = 30).
(DOCX)

S2 Fig Effect of RNA interference on the survival rate of *Nephotettix cincticeps* adult males (see Fig 3B). Survival rates of adult males. Two independent experiments were performed. Fig 3B is the sum of these data. Ds*NcSP75* (black), ds*EGFP* (gray), and untreated controls (white). In the 1st experiment (solid line), Ds*NcSP75* (n = 20), ds*EGFP* (n = 20), and untreated controls (n = 20). In the 2nd experiment (dashed line), Ds*NcSP75* (n = 20), ds*EGFP* (n = 20), and untreated controls (n = 17).
(DOCX)

S3 Fig Effect of RNA interference on the survival rate of *Nephotettix cincticeps* adult males fed an artificial diet (see Fig 4). Survival rates of adult males fed an artificial diet. Two independent experiments were performed. Fig 4 is the sum of these data. Ds*NcSP75* (black) and ds*EGFP* (gray). In the 1st experiment (solid line), Ds*NcSP75* (n = 32) and ds*EGFP* (n = 35). In the 2nd experiment (dashed line), Ds*NcSP75* (n = 35) and ds*EGFP* (n = 34).
(DOCX)

S4 Fig Effect of RNA interference of *NcSP75* nymphs hatching from eggs laid by adult females. (A) Survival rates of females. The conditions were the same as those described in Fig 2B. No significant differences were found. (B) The mean number of hatched nymphs from each female per day is indicated by a dot. The number of mated females is shown in parentheses. The mean number of hatched nymphs is indicated by a diamond symbol. No nymph hatched from eggs laid by four ds*NcSP75-treated* and two ds*EGFP-treated* females. Different letters next to the diamond symbols indicate significant differences (p < 0.01).
(DOCX)

S1 Table The lifespan and duration of each nymphal stage after dsRNA injection. (DOCX)

S2 Table The lifespan of adult males in rice plants after dsRNA injection. (DOCX)
