# Supplementary material for: The green rice leafhopper, Nephotettix cincticeps (Hemiptera: Cicadellidae), salivary protein NcSP75 is a key effector for successful phloem ingestion
Source: PLoS One. 2018 Sep 5;13(9):e0202492. doi: 10.1371/journal.pone.0202492 (PMC6124752; doi:10.1371/journal.pone.0202492)
Supplement: S1 Fig — Survival rates of adult females. Two independent experiments were performed. Fig 2B is the sum of these data. DsNcSP75 (black), dsEGFP (gray), and untreated controls (white). In the 1st experiment (solid line), DsNcSP75 (n = 41), dsEGFP (n = 29), and untreated controls (n = 29). In the 2nd experiment (dashed line), DsNcSP75 (n = 30), dsEGFP (n = 29), and untreated controls (n = 30). (DOCX) [file pone.0202492.s001.docx]

|  |
| --- |

**S1 Fig. Effect of RNA interference on the survival rate of *Nephotettix cincticeps* adult females (see Fig 2B).**

Survival rates of adult females. Two independent experiments were performed. Fig. 2B is the sum of these data. Ds*NcSP75* (black), ds*EGFP* (gray), and untreated controls (white). In the 1st experiment (solid line), Ds*NcSP75* (n = 41), ds*EGFP* (n = 29), and untreated controls (n = 29). In the 2nd experiment (dashed line), Ds*NcSP75* (n = 30), ds*EGFP* (n = 29), and untreated controls (n = 30).
