# Supplementary material for: The green rice leafhopper, Nephotettix cincticeps (Hemiptera: Cicadellidae), salivary protein NcSP75 is a key effector for successful phloem ingestion
Source: PLoS One. 2018 Sep 5;13(9):e0202492. doi: 10.1371/journal.pone.0202492 (PMC6124752; doi:10.1371/journal.pone.0202492)
Supplement: S3 Fig — Survival rates of adult males fed an artificial diet. Two independent experiments were performed. Fig 4 is the sum of these data. DsNcSP75 (black) and dsEGFP (gray). In the 1st experiment (solid line), DsNcSP75 (n = 32) and dsEGFP (n = 35). In the 2nd experiment (dashed line), DsNcSP75 (n = 35) and dsEGFP (n = 34). (DOCX) [file pone.0202492.s003.docx]

|  |
| --- |

**S3 Fig. Effect of RNA interference on the survival rate of *Nephotettix cincticeps* adult males fed an artificial diet (see Fig 4).**

Survival rates of adult males fed an artificial diet. Two independent experiments were performed. Fig. 4 is the sum of these data. Ds*NcSP75* (black) and ds*EGFP* (gray). In the 1st experiment (solid line), Ds*NcSP75* (n = 32) and ds*EGFP* (n = 35). In the 2nd experiment (dashed line), Ds*NcSP75* (n = 35) and ds*EGFP* (n = 34).
