# Supplementary material for: The green rice leafhopper, Nephotettix cincticeps (Hemiptera: Cicadellidae), salivary protein NcSP75 is a key effector for successful phloem ingestion
Source: PLoS One. 2018 Sep 5;13(9):e0202492. doi: 10.1371/journal.pone.0202492 (PMC6124752; doi:10.1371/journal.pone.0202492)
Supplement: S4 Fig — (A) Survival rates of females. The conditions were the same as those described in Fig 2B. No significant differences were found. (B) The mean number of hatched nymphs from each female per day is indicated by a dot. The number of mated females is shown in parentheses. The mean number of hatched nymphs is indicated by a diamond symbol. No nymph hatched from eggs laid by four dsNcSP75-treated and two dsEGFP-treated females. Different letters next to the diamond symbols indicate significant differences (p < 0.01). (DOCX) [file pone.0202492.s004.docx]

| (A) (B)   |
| --- |

**S4 Fig. Effect of RNA interference of *NcSP75* nymphs hatching from eggs laid by adult females.**

(A) Survival rates of females. The conditions were the same as those described in Fig 2B. No significant differences were found. (B) The mean number of hatched nymphs from each female per day is indicated by a dot. The number of mated females is shown in parentheses. The mean number of hatched nymphs is indicated by a diamond symbol. No nymph hatched from eggs laid by four ds*NcSP75-treated* and two ds*EGFP-treated* females. Different letters next to the diamond symbols indicate significant differences (p < 0.01).
