# Supplementary material for: The green rice leafhopper, Nephotettix cincticeps (Hemiptera: Cicadellidae), salivary protein NcSP75 is a key effector for successful phloem ingestion
Source: PLoS One. 2018 Sep 5;13(9):e0202492. doi: 10.1371/journal.pone.0202492 (PMC6124752; doi:10.1371/journal.pone.0202492)
Supplement: S1 Table — (DOCX) [file pone.0202492.s005.docx]

**S1 Table. The lifespan and duration of each nymphal stage after dsRNA injection**

|  |  | **Lifespan after**  **dsRNA**  **injection (days, means ± SE)** |  | **Developmental stages (days, means ± SE)** | | | |
| --- | --- | --- | --- | --- | --- | --- | --- |
|  | ***n*** |  |  | **3rd instar** | **4th instar** | **5th instar** | **Adult**  **(until death)** |
| **ds*NcSP75*** | **31** | **15.45 ± 1.50**** |  | **3.50 ± 0.14**** **(26)** | **7.22 ± 0.44**** **(18)** | **12.67 ± 1.20**** **(3)** | **1.67 ± 0.67*** **(3)** |
| **ds*EGFP*** | **29** | **30.59 ± 3.94** |  | **3.61 ± 0.10**** **(23)** | **3.67 ± 0.17 (21)** | **5.40 ± 0.20 (20)** | **29.05 ± 3.43 (20)** |
| **Untreated control** | **30** | **39.10 ± 3.57** |  | **2.71 ± 0.09 (28)** | **3.39 ± 0.09 (28)** | **5.11 ± 0.16 (27)** | **31.59 ± 3.25 (27)** |

*n*, the numbers of insects used.

Asterisks indicate significant differences in respective periods (*p < 0.05, **p < 0.01).

See Figs 1B and 1C.
