# Supplementary material for: The green rice leafhopper, Nephotettix cincticeps (Hemiptera: Cicadellidae), salivary protein NcSP75 is a key effector for successful phloem ingestion
Source: PLoS One. 2018 Sep 5;13(9):e0202492. doi: 10.1371/journal.pone.0202492 (PMC6124752; doi:10.1371/journal.pone.0202492)
Supplement: S2 Table — (DOCX) [file pone.0202492.s006.docx]

**S2 Table. The lifespan of adult males in rice plants after dsRNA injection**

|  | ***n*** | **Days (means ± SE)** |
| --- | --- | --- |
| **ds*NcSP75*** | **40** | **6.48 ± 0.88**** |
| **ds*EGFP*** | **40** | **20.13 ± 1.67** |
| **Untreated control** | **37** | **18.32 ± 1.98** |

*n*, the numbers of insects used.

Asterisks indicate significant differences in respective adults (**p < 0.01).

See Fig 3B.
